# Synergistic Interaction of Light Alcohol Administration in the Presence of Mild Iron Overload in a Mouse Model of Liver Injury: Involvement of Triosephosphate Isomerase Nitration and Inactivation

**DOI:** 10.1371/journal.pone.0170350

**Published:** 2017-01-19

**Authors:** Wanxia Gao, Jie Zhao, Zhonghong Gao, Hailing Li

**Affiliations:** 1 School of Chemistry and Chemical Engineering, Huazhong University of Science & Technology, Wuhan, P. R. China; 2 Basis medical college, Hubei University of Science and Technology, Xianning, P. R. China; 3 Hubei Key Laboratory of Bioinorganic Chemistry & Materia Medica, Wuhan, P. R. China; University of Kentucky, UNITED STATES

## Abstract

It is well known that iron overload promotes alcoholic liver injury, but the doses of iron or alcohol used in studies are usually able to induce liver injury independently. Little attention has been paid to the coexistence of low alcohol consumption and mild iron overload when either of them is insufficient to cause obvious liver damage, although this situation is very common among some people. We studied the interactive effects and the underlining mechanism of mild doses of iron and alcohol on liver injury in a mouse model. Forty eight male Kunming mice were randomly divided into four groups: control, iron (300 mg/kg iron dextran, i.p.), alcohol (2 g/kg/day ethanol for four weeks i.g.), and iron plus alcohol group. After 4 weeks of treatment, mice were sacrificed and blood and livers were collected for biochemical analysis. Protein nitration level in liver tissue was determined by immunoprecipitation and Western blot analysis. Although neither iron overload nor alcohol consumption at our tested doses can cause severe liver injury, it was found that co-administration of the same doses of alcohol and iron resulted in liver injury and hepatic dysfunction, accompanied with elevated ratio of NADH/NAD^+^, reduced antioxidant ability, increased oxidative stress, and subsequent elevated protein nitration level. Further study revealed that triosephosphate isomerase, an important glycolytic enzyme, was one of the targets to be oxidized and nitrated, which was responsible for its inactivation. These data indicate that even under low alcohol intake, a certain amount of iron overload can cause significant liver oxidative damage, and the modification of triosephosphate isomerasemight be the important underlining mechanism of hepatic dysfunction.

## Introduction

Alcohol consumption is prevalent in societies and long-term alcohol consumption leads to alcoholic liver disease ranging from initial steatosis to cirrhosis. The pathogenesis of alcoholic liver disease is a sophisticated process and iron is thought to play a key role in the progression of alcoholic liver disease [1]. Iron is an essential trace element, which is very important in living body. However, excessive iron and alcohol are associated with higher risk of liver disease and hepatocellular carcinoma [2]. Interactions between moderate levels of alcohol and iron are of great medical importance. With the improvement of living standards, alcohol consumption often accompanies with iron overload in modern life. Firstly, alcohol is one of the most frequently abused substances by humans, and age-associated iron accumulation was found in various tissues [3]. Secondly, the process of alcohol consumption is always accompaniedwith eating lots of red meat, which contains large amount of easily absorbed form of iron, leading to higher risk of iron overload[4].

The research on the combined harmful effect of alcohol and iron on health has long history. Excessive iron is found to be accumulated in alcoholic liver disease and the progression of alcoholic liver disease is promoted by iron supplementation [5]. Moreover, people with hereditary hemochromatosis who consume alcohol have increased risk of cirrhosis than those who don’t [6]. There is also evidence that iron and alcohol may promote synergistic hepatic mutagenesis [7]. However, most studies are focused on the combined harmful effect on health of iron and alcohol in extremely high range of doses which are able to individually induce liver injury, and little has been known on combined effect of mild doses of iron and alcohol on liver damage. In fact, long term lower alcohol intake under mild iron overload may have much epidemiologic significance.

Iron is an important attributer in alcoholic liver disease progression and the regulator proteins involved in the iron metabolism are heavily influenced by alcohol [1]. It is generally accepted that reactive oxygen species are associated with liver injury regardless of being caused by alcohol or iron [8, 9]. It is also suggested that in the presence of iron, high levels of reduced form of nicotinamide-adenine dinucleotid (NADH) caused by alcohol metabolism is able to promote the formation of H_2_O_2_ [10], and even worse, excess iron can promote the generation of hydroxyl radicals through Fenton reaction. Alcohol can also induce the expression of inducible nitric oxide synthase (iNOS), leading to the increase of nitric oxide (NO) production [11, 12]. Excessive production of NO will interact with superoxide anion to form the highly reactive species, peroxynitrite anion [13]. NO is a major source of reactive nitrogen species (RNS). Reactive oxygen species (ROS) and RNS can cause liver cell damage by inducing inflammation, necrosis, apoptosis, etc.,through modification of lipids, proteins, and DNA [13].

Proteins are among the main targets for ROS and RNS assault. From this point of view, we attempted to find out a direct link between oxidative stress and cell dysfunction. Triosephosphate isomerase (TIM) is one of the most abundant cytoplasmic proteins and an important glycolytic enzyme that catalyzes the interconversion of dihydroxyacetone phosphate and DL-glyceraldehyde 3-phosphate. TIM is easy to be attacked by ROS and RNS [14]. Modifications of TIM would lead to a metabolic block in the glycolytic pathway and result in elevated concentration of dihydroxyacetone phosphate, which are diverted towards fatty acid synthesis [15, 16]. Though oxidative/nitrative stress is well known to play an important role in liver injury, little is known about the joint impact of low dose- iron and alcohol on the extent of posttranslational modifications of hepatic proteins or the underlying mechanism linking between modifications of the glycolytic enzyme and hepatic dysfunction.

This study was designed to examine the liver injury under light iron overload and mild dose of alcohol administration, and also aimed to study the mechanisms underlying the condition. Slight-to-severe alcoholic liver injury models were usually established by different doses (2–8 g/kg/day) of alcohol in murine [17, 18]. To avoid the accumulated effects of the individual toxicity on liver, we specifically chose the low dose of alcohol (2 g/kg/day) and iron (300 mg/kg), based on our previous study [19] rather than high dose to investigate the synergistic interaction of them [20].

## Materials and Methods

### Materials and chemicals

Iron dextran, rabbit polyclonal antibody against 3-nitrotyrosine (anti-3-NT), 2,4-dinitrophenylhydrazine, rabbit polyclonal antibody against dinitrophenol, TIM, triethanolamine hydrochloride, NADH, α-glycerophosphate dehydrogenase, DL-glyceraldehyde 3-phosphate solution, N-ethyl-maleimide, N-acetyl-imidazole and butylated hydroxytoluene were purchased from Sigma (St. Louis, MO, USA). Monoclonal anti-3-NT adducts IgG was obtained from Millipore Corp. (Billerica, MA, USA). Chemiluminescence system was purchased from Pierce (Rockford, USA). Detection kits for alanine aminotransferase (ALT), aspartate aminotransferase (AST), glutathione peroxidase (GPx), glutathione (GSH), catalase (CAT), superoxide dismutase (SOD) and nitric oxide were gained from Nanjing Jiancheng Bioengineering Research Institute (Nanjing, China). NAD(H) kits were purchased from Suzhou Comin Biotechnology Co., Ltd. (Suzhou, China). Rabbit polyclonal antibody against TIM and protein A/G agarose were purchased from Santa Cruz Biotechnology (Santa Cruz, CA, USA). Horseradish peroxidase-conjugated goat anti-rabbit IgG and anti-mouse IgG was obtained from Thermo Fisher (Rockford, USA). All other reagents and chemicals were of analytical grade and purchased from a local reagent retailer.

### Animals and reatments

This study was carried out in strict accordance with the recommendations in the Guide for the Care and Use of Laboratory Animals of the National Institutes of Health. The protocol was approved by the Institutional Animal Care and Use Committee of Huazhong University of Science and Technology. Forty-eight male Kunming mice (18–22 g, 4 weeks old) were purchased from Hubei Research Center for Laboratory Animals (Permit Number: SCXK2008-0005). The mice were acclimatized at 23±2°C with a 12-h light/dark cycle for 1 week, and then randomly assigned to four groups: control group (C); iron overload group (I) which were administrated by intraperitoneal injection of 5 doses of 60 mg/kg iron-dextran-saline every other day; alcohol group (A) which were given 2 g/kg/day ethanol by intragastric administration for 4 weeks; iron plus alcohol group (IA) which were treated with alcohol as group A after administrating with last dose of iron dextran as group I.

### Tissue collection and preparation

Mice were fasted for 12 h, and then anesthetized with ethyl ether, and blood was collected from postcava. The liver was quickly removed and some liver tissue was cut off and used for pathological examination, and the remaining sample was perfused with 4°C saline to exclude blood cells and snap-frozen in liquid nitrogen, and then stored at -80°C for other biochemical tests. Liver tissue was homogenized manually with 9 vol. of 50 mM Tris-HCl (pH 7.4) containing 150 mM NaCl, 1% Triton X-100 and protease inhibitor cocktail. The homogenate was centrifuged at 10000 g at 4°C and the supernatant was used for subsequent biochemical analyses. The protein content was measured by the Bradford method [21].

### Histological analysis

Pathological examination was processed according to our previous study [22]. Briefly, liver tissues were fixed with 4% buffered paraformaldehyde, and embedded in paraffin. Tissue sections were cut and stained with hematoxylin and eosin (H&E). Then these sections were observed by light microscopy and images were collected by Nikon DS-U3 at magnification 400×.

### Biochemical analysis of serum markers and measurement of serum nitric oxide

Blood samples were kept at 4°C for 1 h to coagulate and then serum was collected by centrifugation at 3500 g for 15 min at 4°C. Serum AST, ALT and nitric oxide concentrations were measured using commercial kits.

### Measurement of hepatic iron contents and NADH/NAD^+^ levels

An appropriate amount of liver was digested by the wet method with a mixture of nitric acid and perchloric acid according to our previous study [22], and hepatic iron concentrations were determined by atomic absorption spectroscopy (AAnalyst300; PerkinElmer, USA). Liver NADH and NAD^+^ were estimated by commercially available kits. Hepatic NADH and NAD^+^ were extracted with the appropriate extraction buffer and mixed with assay buffer, and the absorbance was read at 570 nm. The ratio of NADH/NAD^+^ was calculated based on results of NADH and NAD^+^ concentrations.

### Oxidative stress evaluation

Malondialdehyde (MDA), as the end product of lipid peroxidation, was determined as previously described [23]. The liver MDA concentration was expressed as nmol/mg protein. The total reduced glutathione in the liver tissue were evaluated using a commercial assay kit. Data were expressed as μg/mg protein.

The enzyme activities of glutathione peroxidase and catalase as well as superoxide dismutase were measured using commercial kits. Enzyme activities were expressed as U/mg protein.

### Determination of hepatic TIM activity

Hepatic TIM activity was measured as described previously with slight modifications [24]. Liver was homogenated with 9 vol.of 10 mM Tris-HCl buffer (pH 7.4) containing 150 mM KCl, 1 mM EDTA and 10 mM 2-mercaptoethanol. The reaction mixture contained 100 mM triethanolamine buffer (pH 7.6), 4 mM DL-glyceraldehyde 3-phosphate, 0.2 mM NADH and 1.8 U/mL α-glycerophosphate dehydrogenase and liver homogenate. The catalytic activity of TIM was measured at 25°C by monitoring the changes in absorbance at 340 nm.

### Western blot analysis and immunoprecipitation

The detection of protein oxidation or nitration was performed as we described previously [25]. Briefly, for protein oxidation analysis, one volume of liver homogenate was first treated with 3 volume of 2, 4-dinitrophenylhydrazine (10 mM in 2 M HCl). Then an equal volume of neutralization solution was used to terminate the reaction, which followed by loading buffer. Samples were electrophoresed on SDS-polyacrylamide gel (SDS-PAGE). Direct SDS-PAGE was used to detect protein expression or protein nitration. After being transferred to nitrocellulose membranes and blocked, proteins were incubated with primary antibodies against TIM (1:200), iNOS (1:200), 3-nitrotyrosine (1:1000), actin (1:500), or dinitrophenol (DNP, 1:4000) and followed by a secondary antibody. Specific proteins were identified with enhanced Chemiluminescence (Pierce).

Protein extracts was applied for the immunoprecipitation with rabbit polyclonal antibody against 3-nitrotyrosine and TIM as previously described [26]. Briefly, 500 μg liver homogenate was probed with 2 μg anti-3-NT or anti-TIM antibody for 6 h at 4°C. Protein A/G agarose was used to obtain immune complexes at 4°C for 2 h by centrifugation at 10,000 g for 30 seconds. Immunoprecipitated proteins were dissolved by loading buffer and then analyzed by immunoblotting as described above. The densitometric analysis of blots was performed with Tanon5200 software.

### Reaction of TIM with chemical reagents

To assay the effects of thiols or tyrosine modification on TIM activity, baker's yeast TIM (0.2 mg/mL; Sigma) was pre-incubated with various concentrations of N-ethyl-maleimide or N-acetylimidazole in 50 mM Tris-HCl buffer (pH 7.6) at 37°C for 30 min and then TIM activity was examined as described above.

### Statistical analysis

All data shown were obtained from at least three experiments.Results were expressed as the mean ± standard error of the mean (SEM). One-way analysis of variance (ANOVA) followed by Tukey’s test was used for analysis of 4 groups with normal distribution; if samples were not normally distributed, data were analyzed by Kruskal–Wallis test (SPSS 19.0 software package). A probability value of 0.05 was considered to be statistically significant.

## Results

### Liver function and histological analysis

**Fig 1:**
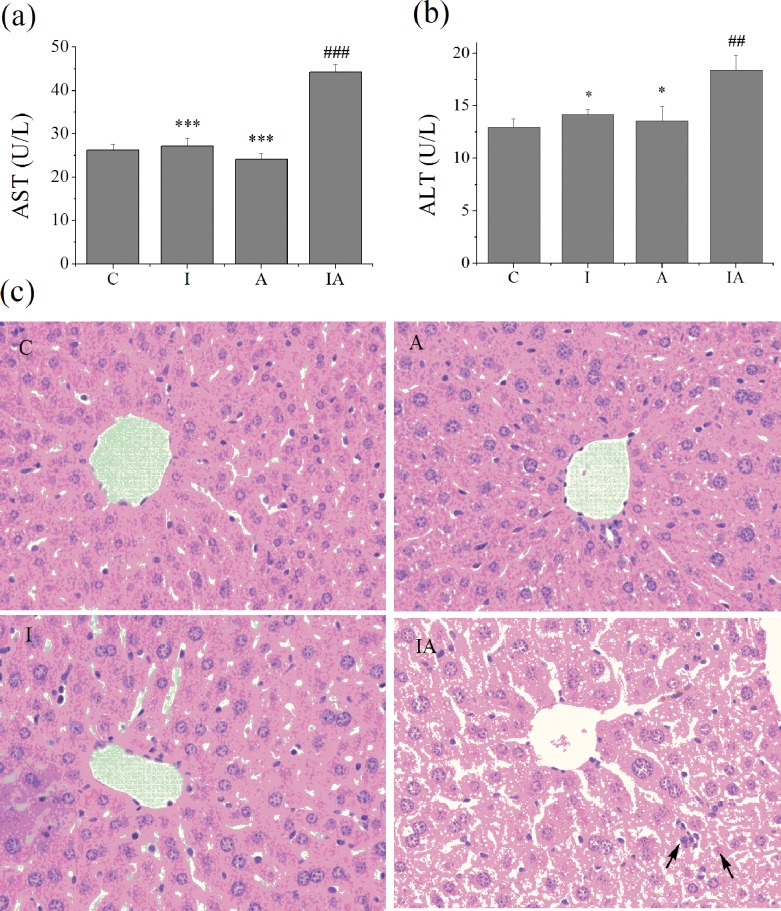
Biochemical indicators of liver function in serum and histological changes in the liver. (a) Serum aspartate aminotransferase (AST) activity, (b) alanine aminotransferase (ALT) activity, and (c) the *arrows* showed karyorrhexis and cytolysis necrosis in fixed liver tissue sections stained with H&E (magnification 400×). Group C, control; group I, iron 300 mg/kg; group A, ethanol 2 g/kg/day; group IA, iron 300 mg/kg + ethanol 2 g/kg/day. Values are expressed as means±SEM, ^##^p<0.01, ^###^p<0.001 vs. groupC; ^*^p<0.05, ^***^p<0.001 vs. groupIA.

Liver injury was evaluated by serum AST and ALT activities. Compared with the controls, there was significant increase in both enzyme activities in group IA but no significant differences in group I or group A, as shown in Fig 1A and 1B. Liver damage was further confirmed by histological examination (Fig 1C). Control mice livers exhibited no abnormal morphological change. The liver cell of group I mice showed slight edema and few vacuoles. In group A, except for mild edema, the hepatic structures were completely normal. The livers of group IA showed significant lesion, characterized by obvious cell edema, steatosis, karyorrhexis and focal cytolysis necrosis in liver.

### Hepatic iron contents and NADH/NAD^+^ ratios of different treated mice

**Fig 2:**
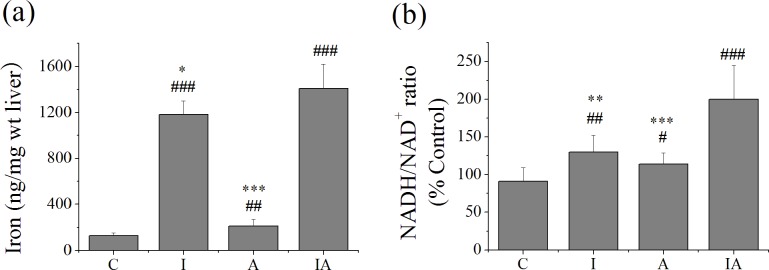
Hepatic iron contents and NADH/NAD^+^ ratio in different treated mice. (a) Liver iron contents were determined by atomic absorption spectroscopy. (b) NADH/NAD^+^ ratios were determined as the total NADH contents divided by total NAD^+^ levels. Group C, control; group I, iron 300 mg/kg; group A, ethanol 2 g/kg/day; group IA, iron 300 mg/kg + ethanol 2 g/kg/day. Values are expressed as means±SEM, ^#^p<0.05, ^##^p<0.01, ^###^p<0.001 vs. groupC; ^*^p<0.05,^**^p<0.01, ^***^p<0.001 vs. groupIA.

Iron is a prooxidative hepatotoxicity factor. In comparison with the controls, liver iron contents were greatly increased in group A and especially in group I. Hepatic iron levels were further promoted in group IA compared with each single group (Fig 2A). Meanwhile, the ratio of NADH/NAD^+^, reflecting the levels of glycolysis and lipid metabolism, was increased in the liver of different treated groups. The greatest increase of NADH/NAD^+^ ratio was found in group IA (Fig 2B).

### Oxidative stress in the livers of different treated mice

**Fig 3:**
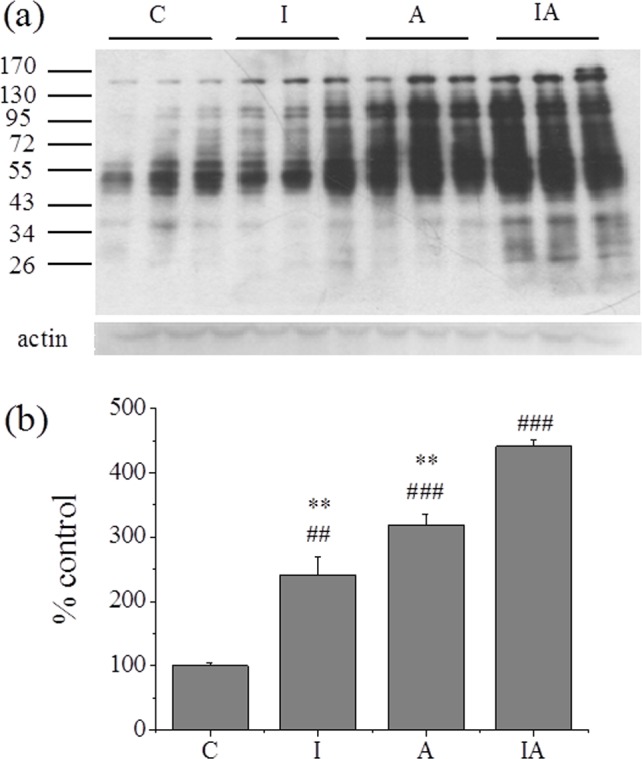
Total oxidative status of hepatic protein in different treated groups. (a) Protein carbonylation of liver, and (b) the corresponding densitometric analysis. Normal values were set to 100%, with which other values were compared. Group C, control; group I, iron 300 mg/kg; group A, ethanol 2 g/kg/day; group IA, iron 300 mg/kg + ethanol 2 g/kg/day. Values are expressed as means±SEM, obtained from nine mice of each group. All visible bands were quantified. ^##^p<0.01, ^###^p<0.001 vs. groupC; ^**^p<0.01 vs. groupIA.

Oxidative stress results from an imbalance between the generations of oxygen derived radicals and the organism's antioxidant potential. Results of hepatic MDA levels, thiol contents, and antioxidant enzyme activities as well as protein oxidation status were shown in Table 1 and Fig 3. Both group I and group A showed elevated levels of liver MDA and protein carbonyl. Group IA exhibited the most prominent increase in the levels of MDA and protein oxidation. The antioxidant enzyme activity, such as SOD, CAT and GPx were decreased with different degrees regardless of in group I or in group A, and they all showed the most dramatic drops in group IA. One paradoxical result was that the thiol contents increased rather than decreased with the decrease in activity of antioxidant enzymes.

**Table 1:** Lipid peroxidation, total GSH content and antioxidant enzymes activities in the liver of different groups.

	C	I	A	IA
MDA (nmol/mg protein)	0.407±0.013	0.573±0.015^###^^,^^***^	0.461±0.009^##^^,^^***^	0.695±0.019^###^
GSH (μg/ mg protein)	4.8±0.3	6.8±0.4^##^	8.4±0.6^###^^,^^*^	6.7±0.3^##^
CAT (U/mg protein)	103.1±6	95.2±2.5^***^	92.9±4^***^	68.6±5^###^
SOD (U/mg protein)	304.3±16.9	247.4±5.2^##^	256.4±7.1^#^	241.7±6^###^
GPx (U/mg protein)	239.3.6±6.9	220.2±6.1^***^	229.2±6.7^***^	164.4±3.7^###^

Group C, control; group I, iron 300 mg/kg; groupA, ethanol 2 g/kg/day; groupIA, iron 300 mg/kg + ethanol 2 g/kg/day. Values are expressed as means±SEM

^#^p<0.05

^##^p<0.01

^###^p<0.001 vs. groupC

*p<0.05

***p<0.001 vs. groupIA.

### Nitrative stress in the livers of different treated mice

**Fig 4:**
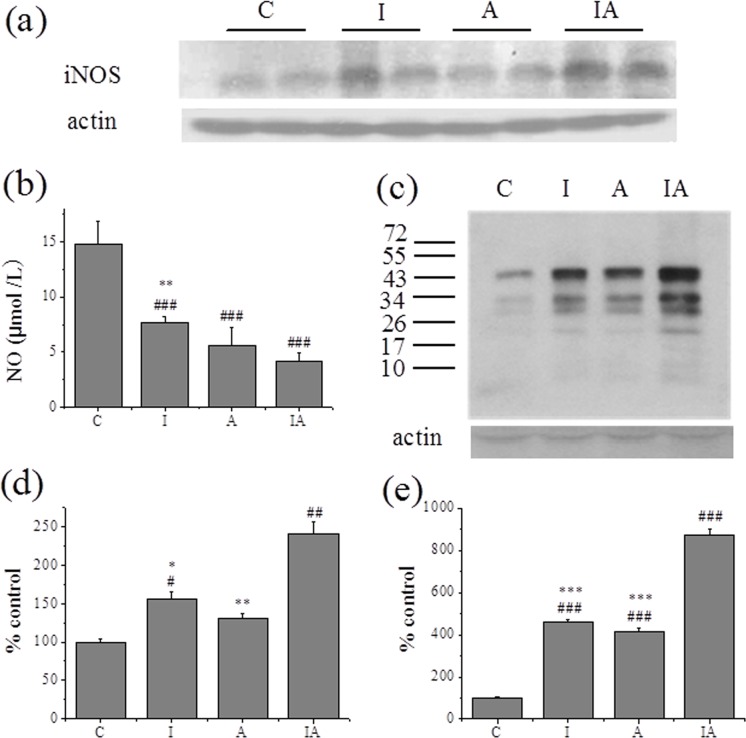
Nitrative stress in the liver of different treated groups. (a) Hepatic iNOS expression, (b) serum NO content, and (c) hepatic protein nitration level. (d) and (e) the corresponding densitometric analysis of iNOS expression and total nitration status of liver protein. The respective control values were set to 100%, to which the other groups’ values were compared. Group C, control; group I, iron 300 mg/kg; group A, ethanol 2 g/kg/day; group IA, iron 300 mg/kg + ethanol 2 g/kg/day. Values are expressed as means±SEM, ^#^p<0.05, ^##^p<0.01, ^###^p<0.001 vs.groupC; ^*^p<0.05, ^**^p<0.01, ^***^p<0.001 vs. groupIA.

To further study the possible role of nitrative stress in liver lesion, we measured serum NO, hepatic iNOS expression and nitrotyrosine levels in different treated groups. As shown in Fig 4A, there was gradual elevation of iNOS expression in group I, and group IA. However, the serum NO levels were declined sharply in group I and group A, particularly in group IA (Fig 4B). Consistent with the results of iNOS expression, marked increase of total level of protein tyrosine nitration was found in groupI and group A, especially in group IA (Fig 4C, 4D and 4E).

### Hepatic TIM catalytic activity, expression, oxidation and nitration in different treated mice

**Fig 5:**
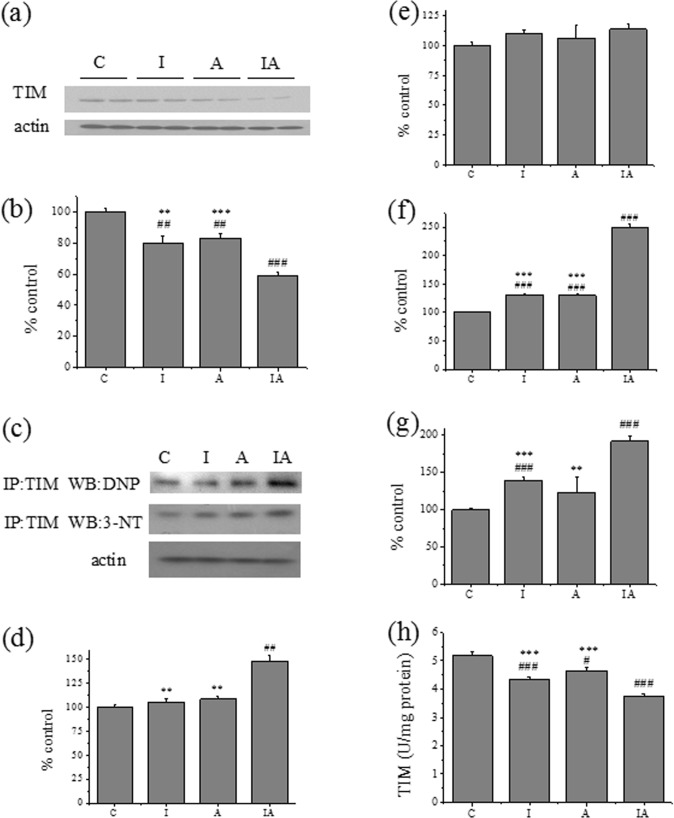
TIM expression, oxidation, nitration and catalytic activity in livers of different treated groups. (a) Hepatic TIM expression and (b) the corresponding densitometric analysis. (c) Oxidation and nitration status of hepatic TIM. (d) The corresponding densitometric analysis of TIM oxidation. (e) The corresponding densitometric analysis of nitration. (f) The ratio of TIM oxidation to total TIM expression. (g) The ratio of TIM nitration to total TIM expression. (h) TIM activity. Equal amounts of protein were immunoprecipitated by anti-TIM antibody, and immunoprecipitates were analyzed for oxidation and nitration status of TIM by Western blot. The respective control values were set to 100%, to which the other groups’ values were compared. Group C, control; group I, iron 300 mg/kg; group A, ethanol 2 g/kg/day; group IA, iron 300 mg/kg + ethanol 2 g/kg/day. Values are expressed as means±SEM, ^#^p<0.05, ^##^p<0.01, ^###^p<0.001 vs. group C; ^**^p<0.01, ^***^p<0.001 vs. group IA.

Immunoprecipitation and immunoblotting were used to measure the expression and modifications of hepatic TIM under the administration of iron and alcohol. We found that TIM expression was decreased in group I and group A, especially in group IA (Fig 5A and 5B). Compared with control group, carbonyl levels and tyrosine nitration levels of TIM were slightly increased in group I and A, but significantly increased in group IA (Fig 5C to 5G); the ratio of oxidized or nitrated TIM to total TIM was even more significantly enhanced in all treated groups, particularly in group IA (Fig 5F and 5G). TIM activities of different treated group were also measured. As expected, the activity of TIM was decreased with the decreasing of TIM expression and the increasing of its oxidation and nitration in group I, A and IA, and the activity of TIM in group IA was the lowest (Fig 5C–5H).

### Modification of TIM *in vitro*

**Fig 6:**
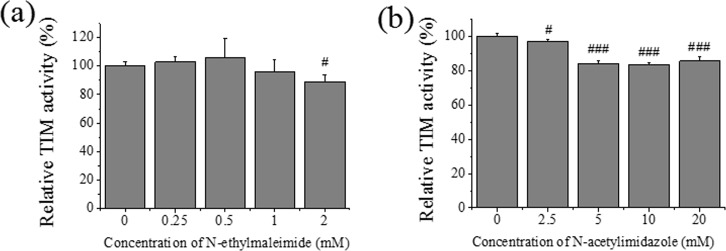
Effects of chemical regents on TIM activity. (a) Effects of N-ethyl-maleimide on TIM activity. (b) Effects of 1-acetyl-imidazole on TIM activity. ^#^P< 0.05, ^##^P< 0.01, ^###^p < 0.001.

To further investigate the potential contribution of thiol oxidation and nitrative modification on TIM activity, N-ethyl-maleimide and N-acetyl-imidazole were used to modify cysteine residues and tyrosine residues, respectively. As shown in Fig 6A and 6B, N-ethyl-maleimide caused the decrease of TIM activity at high concentration, whereas N-acetyl-imidazolein inactivated TIM in a dose-dependent manner. It meant that, besides oxidation, nitration of TIM was also responsible for the enzyme inactivation.

## Discussion

There are many animal models to study the synergistic effect of iron and alcohol. Simultaneous administration of iron and large dosage ethanol to establish rat models of hepatic fibrosis or cirrhosis has been reported [27, 28]. Tan et al. found mild iron overload could enhance alcoholand obesityinduced liver injury in mice [29]. In this study, we established the animal model specifically using mild levels of iron and alcohol: one is for avoiding the additive effect of individual toxicity at high doses, and the other is for closely mimicking the physiological conditions commonly seen in some people’s daily life. Under such circumstance, we could find out whether a combination of the two safe-seeming doses is harmful. Administrating alcohol with different doses, duration and times to mice would cause different degrees of liver injury. Low dose of alcohol (2 g/kg/day) showed no histopathological difference compared with control rats [30]. In our previous studies, we found different amount of iron (150, 300, 500 mg/kg) caused slight-to-severe rat liver injury [19]. To embody the interaction of iron and alcohol, we specially chose 300 mg/kg iron and 2 g/kg/day alcohol to establish the animal model in the present study. Histopathology examination showed that only co-exposure group led to liver steatosis and necrosis, suggesting the liver injury is intensified by the combination use of alcohol and iron, even each separateseems to be safe.Usually, researchers used higher doses of iron or alcohol to investigate the synergic toxicities [27–29]. Our established animal model is a complement to the previous studies on the synergistic effect of iron and alcohol.

As the main organ of medicines transformation metabolism, liver is the inevitable target for iron or alcohol toxicity. Ethanol is metabolized mainly by alcoholic dehydrogenase and cytochrome P4502E1 [31]. Alcohol dehydrogenase and acetaldehyde dehydrogenase are responsible for complete metabolism of mild dose of ethanol [32]. NAD^+^ is the coenzyme of both alcohol dehydrogenase and acetaldehyde dehydrogenase. There are also evidences that chronic and acute ethanol consumption increases the NADH/NAD^+^ ratio [33–36]. The elevated NADH/NAD^+^ ratio results in an imbalance in the redox state of the cell contributing to increased ROS production [37]. In addition, increased ratio of NADH/NAD^+^ is also suggested to contribute to the pathogenesis of fatty liver in part by modulating fatty acid metabolism [38]. In our experiment, NADH/NAD^+^ ratio were increased in alcohol group consistent with other studies [35, 36], besides, we found iron overload further facilitated the NADH/NAD^+^ ratio. High level of NADH was found to facilitate the formation of H_2_O_2_, and the existence of iron would catalyze the generation of free radicals through Fenton reaction [10].Taken together, the results could have implications for better understanding of how a strong synergy of ROS was generated by iron and alcohol.

In view of the differential effects of iron and alcohol on ROS production, combined exposure of ethanol and iron would greatly expedite the generation of ROS. As expected, group IA showed the greatest increase of MDA levels among all groups (Table 1). Antioxidant system was suggested to be impacted by overproduction of lipid peroxidation [39, 40]. We also found that the three antioxidant enzymes (SOD, CAT, and GPx) were decreased more significantly in group IA than other two treated groups (Table 1), indicating the most severe oxidative stress in group IA. However, total hepatic GSH contents were increased instead of being reduced in other three groups in contrast with the control group, which was suggested to be an adaptive response mechanism that counteracted overproduction of ROS [41]. Compared with group I and group A, the increase of GSH level in group IA is slower (Table 1). In addition, we found the highest protein carbonyl level in group IA (Fig 3). Together, our results indicated that even under seemingly safe doses of alcohol, iron overload would cause amplified liver oxidative stress.

Inducible nitric oxide synthase expression is increased and thought to be required in alcoholic liver disease [11, 42]. Nitric oxide catalyzed by iNOS plays an important role in the development of liver injury [11, 12]. NO can interact with superoxide anion or other ROS to form peroxynitrite or other reactive nitrogen species even at low concentration, leading to irreversible modification of proteins, lipids and nucleic acids [12]. By means of immunoprecipitation and immunoblotting, we found that proteins nitration level wassignificantly enhanced in group IA, and the molecular weight of nitrated proteins were in the range of 25 to 55 kDa (Fig 4C). This result was consistent with the result of iNOS expression (Fig 4A). However, the content of NO was decreased in group IA(Fig 4B). The increased nitrated proteins by RNS probably reflects the consumption of NO by superoxide anion, which then contributed to the up-regulation of iNOS in group IA by negative feedback [43].Protein tyrosine nitration is a kind of posttranslational modification when tissues under nitrative stress. It was reported that nitration of mitochondrial proteins led to their inactivation and mitochondrial dysfunction[44], and a recent report revealed that nitration of Y257 in sirtuin 6 inhibited its function [45]. Nitration of TIM at Y165 and Y209 has been demonstrated to induce a decrease in its activity [46]. Based on measured results, we tended to study a direct link between posttranslational modifications and the function of this glycolytic enzyme involved in the pathology of liver injury.

As a key enzyme in cell metabolism, TIM is one of the most abundant cytoplasmic proteins accounting for approximately 1.5% of total soluble protein [47]. Documented evidence demonstrated TIM was also one of the proteins most nitrotyrosinated in neurodegenerative disease [14, 48, 49]. According to the comparison and analysis from previous proteomics studies [48, 50–52], we speculated that TIM was the susceptive target for oxidative and nitrative modification under the treatment of mild doses of iron and alcohol. Thus, TIM was immunoprecipitated and then detected by Western blot with anti-3-nitrotyrosine and anti-DNP antibodies. We observed that elevated levels of carbonyl and 3-nitrotyrosine of TIM in group IA accompanied with its decreased expression and activity (Fig 5). The decrease in TIM expression probably results from oxidative stress, sinceMorel and Barouki reported that moderate (i.e. non-cytotoxic) oxidative stress specifically down-regulates the expression of various genes [53]. We further demonstrated that tyrosine modification or thiol modification of TIM would cause TIM inactivation (Fig 6A and 6B).

There were evidences that TIM deficiency would lead to deviant lipid metabolism [54, 55]. TIM catalyzes the interconversion of dihydroxyacetone phosphate and DL-glyceraldehyde 3-phosphate which is interconnected to lipid metabolism via triosephosphates. The decrease of TIM activity leads tothe increase of dihydroxyacetone phosphate level [54, 55]. The reduction of dihydroxyacetone phosphate toα-glycerophosphate is coupled with NADH to NAD^+^ conversion andα-glycerophosphate is a substrate for the biosynthesis of glycerolipids [54]. Thus, the increase of NADH/NAD^+^ ratio and the decreased activity of TIM will help promote the diversion toward the synthesis ofα-glycerophosphate, which might lead to lipid abnormalities of pathogenetic significance.

**Fig 7:**
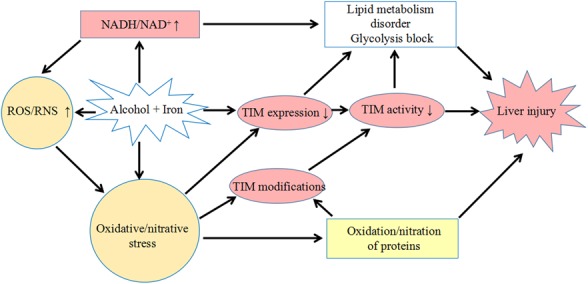
The plausible mechanism of liver injury induced by low doses of alcohol and iron overload. Co-exposure to alcohol and iron led to elevated NADH/NAD^+^ ratio and ROS/RNS, as well as low expression and activity of TIM. Oxidative/nitrative modifications of TIM caused the enzyme inactivity. The disturbed balance of NADH/NAD^+^ ratio and decreased expression and activity of TIM caused by a combination use of iron and ethanol, was hypothesized to favor the abnormalities in energy metabolism and hepatic lipid accumulation associated with liver disease.

In our animal study, we found that when the mice were co-administrated with mild dose of alcohol and iron, thatwould cause significant liver injury, although eachseparatetreatmentseemed relatively safe. The plausible mechanism of liver injury induced by low doses of alcohol under mild iron overload was described in Fig 7. The differential effects of iron and alcohol on generation of ROS as well as NADH made the production of ROS and then RNS overwhelm the antioxidant system, resulting in hepatic protein modification and expression disorder. Both the decrease in the expression and the oxidative/nitrative modifications of TIM resulted in enzyme inactivation, leading to a metabolic block in the glycolytic pathway and disturbance of lipid metabolism, especially when in high level of NADH.

This study has some limitations. For our experiment period was not very long, no severe histological damage was observed. A more severe hepatic injury and more findings would be obtained by prolong the time of combined action of iron and alcohol. Only TIM was investigated in this study, and in fact a number of proteins were modified under oxidative stress that might involve in hepatic injury. Follow up work will more carefully decipher the mechanisms linking between modifications of hepatic proteins and liver injury.

In conclusion, this study provided evidence for the increased risk of liver injury by combination intake of alcohol and iron, even each separate seems to be safe. TIM is one of the susceptible enzymes to be nitrated and oxidized, and the oxidative/nitrative modification of TIM is partially contributing to liver dysfunction.

## References

[1] Mueller S, Rausch V.The role of iron in alcohol-mediated hepatocarcinogenesis. Adv Exp Med Biol. 2015; 815:89–112. 10.1007/978-3-319-09614-8_6 25427903

[2] Stål P, Hultcrantz R. Iron increases ethanol toxicity in rat liver. J Hepatol. 1993; 17:108–115. 8445209 10.1016/s0168-8278(05)80530-6

[3] Brewer GJ. Risks of copper and iron toxicity during aging in human. Chem Res Toxicol. 2009; 23:319–326. 10.1021/tx900338d 19968254

[4] Valencia-Martín JL, Galán I, Rodríguez-Artalejo F. The association between alcohol consumption patterns and adherence to food consumption guidelines. Alcohol: Clin Exp Res. 2011; 35:2075–2081. 21848958 10.1111/j.1530-0277.2011.01559.x

[5] Kohgo Y, Ohtake T, Ikuta K, Suzuki Y, Hosoki Y, Saito H, Kato J. Iron accumulation in alcoholic liver diseases. Alcohol: Clin Exp Res. 2005; 29:189S–193S. 16344607 10.1097/01.alc.0000189274.00479.62

[6] Petersen DR. Alcohol, iron-associated oxidative stress, and cancer. Alcohol. 2005; 35:243–249. 10.1016/j.alcohol.2005.03.013 16054986

[7] Asare GA, Bronz M, Naidoo V, Kew MC. Synergistic interaction between excess hepatic iron and alcohol ingestion in hepatic mutagenesis. Toxicology. 2008; 254:11–18. 10.1016/j.tox.2008.08.024 18852013

[8] Koskenkorva-Frank TS, Weiss G, Koppenol WH, Burckhardt S. The complex interplay of iron metabolism, reactive oxygen species, and reactive nitrogen species: insights into the potential of various iron therapies to induce oxidative and nitrosative stress. Free Radic Biol Med.2013; 65:1174–1194. 10.1016/j.freeradbiomed.2013.09.001 24036104

[9] Qin LY, Crews FT. NADPH oxidase and reactive oxygen species contribute to alcohol-induced microglial activation and neurodegeneration. J Neuroinflammation. 2012; 9:5–9. 10.1186/1742-2094-9-5 22240163 PMC3271961

[10] Kukiełka E, Cederbaum AI. NADH-dependent microsomal interaction with ferric complexes and production of reactive oxygen intermediates. Arch Biochem Biophys.1989; 275:540–550. 2556968 10.1016/0003-9861(89)90400-1

[11] Tirapelli LF, Batalhão ME, Jacob-Ferreira AL, Tirapelli DP, Carnio EC, Tanus-Santos JE, Queiroz RH, Uyemura SA, Padovan CM, Tirapelli CR. Chronic ethanol consumption induces histopathological changes and increases nitric oxide generation in the rat liver. Tissue Cell.2011; 43:384–391. 10.1016/j.tice.2011.08.003 21930289

[12] Venkatraman A, Shiva S, Wigley A, Ulasova E, Chhieng D, Bailey SM, Darley-Usmar VM. The role of iNOS in alcohol-dependent hepatotoxicity and mitochondrial dysfunction in mice. Hepatology. 2004; 40:565–573. 10.1002/hep.20326 15349894

[13] Diesen DL, Kuo PC. Nitric oxide and redox regulation in the liver: Part I. General considerations and redox biology in hepatitis. J Surg Res. 2010; 162: 95–109. 10.1016/j.jss.2009.09.019 20444470 PMC2885581

[14] Butterfield DA, Reed TT, Perluigi M, De Marco C, Coccia R, Keller JN, Markesbery WR, Sultana R. Elevated levels of 3-nitrotyrosine in brain from subjects with amnestic mild cognitive impairment: implications for the role of nitration in the progression of Alzheimer's disease. Brain Res. 2007; 1148:243–248. 10.1016/j.brainres.2007.02.084 17395167 PMC1934617

[15] Grüning NM, Du D, Keller MA, Luisi BF, Ralser M. Inhibition of triosephosphate isomerase by phosphoenolpyruvate in the feedback-regulation of glycolysis. Open Biol. 2014; 4:130232 10.1098/rsob.130232 24598263 PMC3971408

[16] Lee WH, Choi JS, Byun MR, Koo KT, Shin S, Lee SK, Surh YJ. Functional inactivation of triosephosphate isomerase through phosphorylation during etoposide-induced apoptosis in HeLa cells: Potential role of Cdk2. Toxicology. 2010; 278:224–228. 10.1016/j.tox.2010.02.005 20149834

[17] Chou CH, Chang YY, Tzang BS, Hsu CL, Lin YL, Lin HW, Chen YC. Effects of taurine on hepatic lipid metabolism and anti-inflammation in chronic alcohol-fed rats. Food Chem. 2012; 135:24–30.

[18] Wu D, Wang X, Zhou R, Yang L, Cederbaum AI. Alcohol steatosis and cytotoxicity: the role of cytochrome P4502E1 and autophagy. Free Radical Biol Med. 2012; 53:1346–1357. 22819980 10.1016/j.freeradbiomed.2012.07.005 PMC3436962

[19] Zhang Y, Huang Y, Deng X, Gao ZH, Li HL. Iron overload-induced rat liver injury: Involvement of protein tyrosine nitration and the effect of baicalin. Eur J Pharmacol.2012; 680:95–101. 10.1016/j.ejphar.2012.01.010 22306240

[20] Tsay J, Yang Z, Ross FP, Cunningham-Rundles S, Lin H, Coleman R, Mayer-Kuckuk P, Doty SB, Grady RW, Giardina PJ, Boskey AL, Vogiatzi MG. Bone loss caused by iron overload in a murine model: importance of oxidative stress. Blood. 2010; 116:2582–2589. 10.1182/blood-2009-12-260083 20554970 PMC2953890

[21] Bradford MM. A rapid and sensitive method for the quantitation of microgram quantities of protein utilizing the principle of protein-dye binding. Anal Biochem. 1976; 72:248–254. 942051 10.1016/0003-2697(76)90527-3

[22] Gao WX, Li XL, Gao ZH, Li HL. Iron increases diabetes-induced kidney injury and oxidative stress in rats. Biol Trace Elem Res. 2014; 160:368–375. 10.1007/s12011-014-0021-9 24996958

[23] Ohkawa H, Ohishi N, Yagi K. Assay for lipid peroxides in animal tissues by thiobarbituric acid reaction. Anal Biochem. 1979; 95:351–358. 36810 10.1016/0003-2697(79)90738-3

[24] Werman MJ, Bhathena SJ. Fructose metabolizing enzymes in the rat liver and metabolic parameters: Interactions between dietary copper, type of carbohydrates, and gender. J Nutr Biochem. 1995; 6:373–379. 12049998 10.1016/0955-2863(95)80005-w

[25] Li XL, Li HL, Lu NH, Gao ZH. Iron increases liver injury through oxidative/nitrative stress in diabetic rats: Involvement of nitrotyrosination of glucokinase. Biochimie. 2012; 94:2620–2627. 10.1016/j.biochi.2012.07.019 22884880

[26] Lu NH, Zhang Y, Li HL, Gao ZH. Oxidative and nitrative modifications of α-enolase in cardiac proteins from diabetic rats. Free Radic Biol Med. 2010; 48:873–881. 10.1016/j.freeradbiomed.2010.01.010 20079428

[27] Devi SL, Viswanathan P, Anuradha CV. Taurine enhances the metabolism and detoxification of ethanol and prevents hepatic fibrosis in rats treated with iron and alcohol.Environ Toxicol Phar. 2009; 27: 120–126. 10.1016/j.etap.2008.09.004 21783929

[28] Tsukamoto H, Horne W, Kamimura S, Niemelä O, Parkkila S, Ylä-Herttuala S, Brittenham GM. Experimental liver cirrhosis induced by alcohol and iron. J Clin Invest. 1995; 96:620 10.1172/JCI118077 7615836 PMC185237

[29] Tan TCH, Crawford DHG, Jaskowski LA, Subramaniam VN, Clouston AD, Crane DI, Bridle KR, Anderson GJ, Fletcher LM. Excess iron modulates endoplasmic reticulum stress-associated pathways in a mouse model of alcohol and high-fat diet-induced liver injury. Lab Invest. 2013; 93: 1295–1312. 10.1038/labinvest.2013.121 24126888

[30] Zhang F, Zhang J, Li Y. Corn oligopeptides protect against early alcoholic liver injury in rats. Food Chem Toxicol. 2012; 50:2149–2154. 10.1016/j.fct.2012.03.083 22504530

[31] Lieber CS, Abittan CS. Pharmacology and metabolism of alcohol, including its metabolic effects and interactions with other drugs. Clin Dermatol.1999; 17:365–379. 10497719 10.1016/s0738-081x(99)00020-6

[32] Klyosov AA, Rashkovetsky LG, Tahir MK, Keung WM. Possible role of liver cytosolic and mitochondrial aldehyde dehydrogenases in acetaldehyde metabolism. Biochemistry. 1996; 35:4445–4456. 10.1021/bi9521093 8605194

[33] Seronello S, Ito C, Wakita T, Choi J. Ethanol enhances hepatitis C virus replication through lipid metabolism and elevated NADH/NAD^+^. J Biol Chem. 2010; 285:845–854. 10.1074/jbc.M109.045740 19910460 PMC2801286

[34] Ma G, Liu Y, Zhang Q, Zhang B, Zhao N, Wang Q, Wang X. Pre-endurance training prevents acute alcoholic liver injury in rats through the regulation of damaged mitochondria accumulation and mitophagy balance. Hepatol Int. 2014; 8:425–435. 10.1007/s12072-014-9529-5 26202644

[35] Ge N, Liang H, Liu Y, Ma AG, Han L. Protective effect of Aplysin on hepatic injury in ethanol-treated rats. Food Chem Toxicol. 2013; 62:361–372. 10.1016/j.fct.2013.08.071 24001440

[36] Cahill A, Cunningham CC, Adachi M, Ishii H, Bailey SM, Fromenty B, Davies A. Effects of alcohol and oxidative stress on liver pathology: the role of the mitochondrion. Alcohol: Clin Exp Res. 2002; 26:907–915. 12068261 PMC2670542

[37] Liang Y, Harris FL, Jones DP, Brown LA. Alcohol induces mitochondrial redox imbalance in alveolar macrophages. Free Radic Biol Med. 2013; 65:1427–1434. 10.1016/j.freeradbiomed.2013.10.010 24140864 PMC3870467

[38] Lieber CS. Alcoholic fatty liver: its pathogenesis and mechanism of progression to inflammation and fibrosis. Alcohol. 2004; 34:9–19. 10.1016/j.alcohol.2004.07.008 15670660

[39] Choi JS, Koh IU, Lee HJ, Kim WH, Song J. Effects of excess dietary iron and fat on glucose and lipid metabolism. J Nutr Biochem. 2013; 24:1634–1644. 10.1016/j.jnutbio.2013.02.004 23643521

[40] Pandanaboina SC, Kondeti SR, Rajbanshi SL, Qunala PN, Pandanaboina S, Pandanaboina MM, Wudayagiri R. Alterations in antioxidant enzyme activities and oxidative damage in alcoholic rat tissues: protective role of the spesia populnea. Food Chem. 2012; 132:150–159. 10.1016/j.foodchem.2011.10.046 26434274

[41] Cederbaum AI, Lu Y, Wu D. Role of oxidative stress in alcohol-induced liver injury. Arch Toxicol. 2009; 83:519–548. 10.1007/s00204-009-0432-0 19448996

[42] McKim SE, Gabele E, Isayama F, Lambert JC, Tucker LM, Wheeler MD, Connor HD, Mason RP, Doll MA, Hein DW, Arteel GE. Inducible nitric oxide synthase is required in alcohol-induced liver injury: studies with knockout mice. Gastroenterology 2003; 125:1834–1844. 14724835 10.1053/j.gastro.2003.08.030

[43] Vaziri ND, Wang XQ. cGMP-mediated negative-feedback regulation of endothelial nitric oxide synthase expression by nitric oxide. Hypertension.1999; 346:1237–1241. 10.1161/01.hyp.34.6.1237 10601124

[44] Abdelmegeed MA, Jang S, Banerjee A, Hardwick JP, Song BJ. Robust protein nitration contributes to acetaminophen-induced mitochondrial dysfunction and acute liver injury. Free Radic Biol Med. 2013; 60:211–222. 10.1016/j.freeradbiomed.2013.02.018 23454065 PMC3680365

[45] Hu S, Liu H, Ha Y, Motamedi M, Gupta MP, Ma JX, Tilton RG, Zhang W. Posttranslational modification of Sirt6 activity by peroxynitrite. Free Radic Biol Med. 2015; 79:176–185. 10.1016/j.freeradbiomed.2014.11.011 25476852 PMC4339438

[46] Tajes M, Eraso-Pichot A, Rubio-Moscardó F, Guivernau B, Ramos-Fernández E, Bosch-Morató M, Guix FX, Clarimón J, Miscione GP, Boada M, Gil-Gómez G, Suzuki T, Molina H, Villà-Freixa J, Vicente R, Muñoz F. Methylglyoxal produced by amyloid-ß peptide-induced nitrotyrosination of triosephosphate isomerase triggers neuronal death in Alzheimer’s Disease. J Alzheimer’s Dis. 2014; 41:273–288. 24614897 10.3233/JAD-131685

[47] Picotti P, Bodenmiller B, Mueller LN, Domon B, Aebersold R. Full dynamic range proteome analysis of S. cerevisiae by targeted proteomics. Cell. 2009; 138: 795–806. 10.1016/j.cell.2009.05.051 19664813 PMC2825542

[48] Castegna A, Thongboonkerd V, Klein JB, Lynn B, Markesbery WR, Butterfield DA. Proteomic identification of nitrated proteins in Alzheimer's disease brain. J Neurochem. 2003; 85:1394–1401. 12787059 10.1046/j.1471-4159.2003.01786.x

[49] Reed TT, Pierce WM Jr, Turner DM, Markesbery WR, Butterfield DA. Proteomic identification of nitrated brain proteins in early Alzheimer’s disease inferior parietal lobule. J Cell Mol Med. 2009; 13:2019–2029. 10.1111/j.1582-4934.2008.00478.x 18752637 PMC2819643

[50] Barreiro E, Rabinovich RA, Marin J, Barberà JA, Gea J, Roca J. Chronic endurance exercise induces quadriceps nitrosative stress in severe COPD patients. Thorax. 2008; 64: 13–19. 10.1136/thx.2008.105163 18835959

[51] Liu B, Tewari AK, Zhang L, Green-Church KB, Zweier JL, Chen YR, He G. Proteomic analysis of protein tyrosine nitration after ischemia reperfusion injury: mitochondria as the major target, BBA-Proteins Proteomics. 2009; 1794: 476–485. 10.1016/j.bbapap.2008.12.008 19150419 PMC2637933

[52] Palamalai V, Darrow RM, Organisciak DT, Miyagi M. Light-induced changes in protein nitration in photoreceptor rod outer segments. Mol Vis. 2006; 12:1543–1551. 17200653

[53] Morel Y, Barouki R. Repression of gene expression by oxidative stress. Biochem J. 1999; 342: 481–496. 10477257 PMC1220487

[54] Orosz F, Olah J, Ovadi J. Triosephosphate isomerase deficiency: facts and doubts. Iubmb Life. 2006; 58: 703–715. 10.1080/15216540601115960 17424909

[55] Schneider AS. Triosephosphate isomerase deficiency: historical perspectives and molecular aspects. Best Pract Res Cl Ha. 2000; 13: 119–140. 10.1053/beha.2000.0061 10916682

